# 

**Published:** 1914-03-28

**Authors:** 


### CONTENTS.

| | PAGE |
|---|---|
| **The State of the Tuberculosis Service...** ... | 693 |
| **Vacant Accommodation in London Workhouses** ... ... ... ... ... ... ... | 694 |
| **Hospital and Institutional News** ... ... ... | 695 |

The Doctor's Wife—The First Thousand—New Chairman of Charing Cross Hospital—Great Gift to Cardiff Medical School—The Jordan Lloyd Memorial at Birmingham—Bradford's Latest Hospital—Women Suffragists as a Source of Income—A Tragic Medical Suicide—Secretarial Appointments in Birmingham—The Hospital Deadlock in Blackpool—The Medical Staff's Contention—New Secretary for the New Hospital for Women—The Punishment of Infirmary Boys—Mr. Runciman on Medical Certificates—The Soldiers' Daughters' Home, Hampstead—The Mistake of Tinkering—The Nelson Hospital's Need for Extension—Coventry Hospital and Typhoid Cases—The Jessop Hospital and Maternity Benefit—The Insurance Act and Women Doctors—An Indian Hospital Administrator—Perched Around a Billiard Table—A Voice for Hospital Pharmacists—The Salaries of Dispensers—A Successful Subsidiary Organisation—The Opening of the Panama Canal—This Week's Drug Market—To Our Readers.

| | PAGE |
|---|---|
| **Comparative Merits of Some Urinary Antiseptics** ... ... ... ... ... ... | 701 |
| A Clue to the Vagaries of Urinary Sepsis. | |
| **Some Recent Novelties in Diagnostic Technique** ... ... ... ... ... ... | 702 |
| **Electricity and Obesity** ... ... ... ... | 703 |
| Treatment by Electrically Excited Exercise. | |
| **Research and Remedies** ... ... ... ... | 704 |

[*See over.*]

### CONTENTS—(*continued*).

| | PAGE |
|---|---|
| **Reports on Hospitals of the United Kingdom**— By SIR HENRY BURDETT, K.C.B., K.C.V.O | 705 |
| Horsham Cottage Hospital. | |
| The Eliot Cottage Hospital, Hayward's Heath. | |
| **The Origin and Evolution of the 18th Century Hospital Movement** ... ... ... ... | 706 |
| **The Non-Panel Movement** ... ... ... ... | 707 |
| Dr. Edwin Smith on its Position and Prospects. | |
| **The Editor's Letter-Box** ... ... ... ... | 709 |
| Anti-Phthisis Activities in Berkshire—Radium in the Treatment of Deafness—Children and Homœopathy—The Production of Braille Books. | |
| **Hospital Co-operation in Hackney** ... ... | 710 |
| **Co-operative Buying for Hospitals** ... ... | 711 |
| A Comparison of Methods in London and New York. | |
| **The Doctors' Wives Association** ... ... ... | 712 |
| **Hospital Architecture and Construction** ... | 713 |
| A Novel King Edward Memorial. | |
| **Round the World** ... ... ... ... ... ... | 714 |
| Fellow-Workers and their Doings. | |
| **National Insurance Act Developments** ... ... | 715 |
| The Interim Report of the Fabian Society. | |
| **Doctors' Wills** ... ... ... ... ... ... ... | 716 |
| **Appointments** ... ... ... ... ... ... ... | 717 |
| **Gifts and Bequests** ... ... ... ... ... ... | 717 |
| **Institutional Needs** ... ... ... ... ... ... | 717 |
| **The National Medical Union (Official)** ... ... | 718 |
| The Resolutions Adopted last Saturday. | |
| The Profession of Medicine. | |
| **The Institutional Worker** ... ... (Suppl.) | 1 |
| The Filing of Out-patient Case Papers. | |
| Questions for March. | |
| Questions Invited. | |
| The Editor's Letter-box. | |
| **Buildings Contemplated** ... ... Suppl. | 3 |

RATES OF SUBSCRIPTION: Twelve months, 6/6; Six months, 4/-; Three months, 2/-, post free to any address in the United Kingdom. Abroad, Twelve months, 8/8; Six months, 5/-; Three months, 2/6, post free.—Address The Subscription Dept., "THE HOSPITAL," The Hospital Building, 28/29 Southampton Street, Strand, London, W.C.

**BERGONIÉ'S OBESITY TREATMENT**

